# Non-Invasive Assessment of Adrenal Activity in the Subterranean Rodent *Ctenomys talarum* in Field and Laboratory Conditions

**DOI:** 10.3390/ani16020234

**Published:** 2026-01-13

**Authors:** Roxana Zenuto, Valentina Brachetta, María Celina Carrizo, María Sol Fanjul, Cristian Eric Schleich

**Affiliations:** Grupo “Ecología Fisiológica y del Comportamiento”, Instituto de Investigaciones Marinas y Costeras (IIMyC), Universidad Nacional de Mar del Plata, Consejo Nacional de Investigaciones Científicas y Técnicas (CONICET), Mar del Plata 7600, Argentina; rzenuto@mdp.edu.ar (R.Z.); celinacarrizo@mdp.edu.ar (M.C.C.); msfanjul@mdp.edu.ar (M.S.F.); cschleic@mdp.edu.ar (C.E.S.)

**Keywords:** stress, cortisol, plasmatic glucocorticoids, fecal metabolites of glucocorticoids, captive conditions, behavior, *Ctenomys talarum*

## Abstract

The endocrine stress response is a valuable tool for evaluating how organisms cope with environmental challenges. However, selecting an appropriate matrix for measuring glucocorticoids (GCs) requires careful consideration of sample quality and accessibility. This study reveals that blood sampling affects plasma cortisol levels in the subterranean rodent *Ctenomys talarum*, with the effect being reversed shortly thereafter. To facilitate a non-invasive approach, an enzyme-linked immunosorbent assay (EIA) that had previously been validated for measuring plasma cortisol in *C. talarum* was evaluated to measure adrenocortical activity by analyzing fecal glucocorticoid metabolites (FGCs). Using this assay, we monitored the stress response during wild capture, transport to captivity, adrenocorticotropic hormone (ACTH) stimulation, and immobilization. This showed that FGC levels accurately reflect adrenal activation in these contexts. We also documented a relationship between reproductive seasonality and FGCs. Finally, we provide evidence for a relationship between adrenal activity and behavior. Our results suggest that when considering plasma GCs for the assessment of acute stress, it is crucial to understand the magnitude and timing of the effects of blood sampling on the stress state of organisms. The validation of FGC measurement in *C. talarum* provides a new option for advancing ecophysiological studies in both the wild and captivity.

## Simple Summary

In nature, animals experience stress in response to challenges such as harsh weather, predators or food shortages. Particularly in vertebrates, the response to these conditions includes the activation of the hypothalamic–pituitary–adrenal axis, releasing glucocorticoid hormones that quickly boost energy levels. Short bursts of this response help animals survive, but prolonged elevation of these hormones can weaken immunity, impede growth and even disrupt reproduction. Researchers studying wildlife often capture and handle animals through immobilization, blood sampling or temporary captivity, which triggers stress in itself. To understand these effects, we studied *Ctenomys talarum*, a subterranean rodent that lives in natural and human-altered grasslands. We found that standard blood collection causes a brief spike in stress hormones, which return to normal soon afterwards. We also validated a non-invasive assay to measure glucocorticoid metabolites in feces. This test detected increases in hormones after handling, captivity, immobilization and also during breeding seasons. Fecal hormone analysis is less invasive and improves animal welfare while still providing reliable data. Adopting these methods will enhance conservation efforts and help to balance scientific objectives with the well-being of wild animals.

## 1. Introduction

In nature, organisms are exposed to various conditions that threaten homeostasis, both physical and psychological, and could be considered stressors [1,2,3]. The associated physiological response is mediated by the activation of the sympathetic–adrenal–medullary (SAM) and the hypothalamic–pituitary–adrenal (HPA) axes [4,5,6]. Short-term stress response is associated with moderate and brief increases in glucocorticoid (GC) levels, leading to the mobilization of energy reserves needed to cope with the stressful condition, and is considered adaptive since it is essential for the survival of the individual. However, it has been observed that repeated or prolonged increases in GCs affect several critical functions such as immunity, reproduction and growth [7,8,9,10]. Due to the role of GCs in maintaining energy balance—both through their involvement in the catabolism of carbohydrates, proteins and lipids, and their anabolic effects during gluconeogenesis—baseline levels are expected to reflect seasonal and daily changes in energy requirements, as well as those resulting from stressful events [5,9]. Consequently, GCs are considered efficient biomarkers for indexing physiological stress, health status, and general physical condition in captive, free-living, and farmed vertebrates (e.g., [2,11,12,13]). Recently, behavior has been proposed to be a third arm of the stress response, linked to the activation of the SAM and HPA axes, but also functioning independently [14]. Variations in behavioral and physiological responses have been associated with how individuals deal with stressful situations, referred to as “coping styles” [15]. In this context, the behavioral response to a stressful situation is not fixed and may depend not only on the personality of the individual but also on the level and responsiveness of the GCs [16].

Monitoring changes in GC levels in wild rodents is not an easy task [17]. Blood samples are typically collected to measure plasma cortisol and/or corticosterone, depending on the species. The technique used to collect blood samples can have different effects on individuals due to capture, immobilization, anesthesia and other factors that could mask the real GC value [18,19]. The amount of plasma obtained and the frequency of sampling are severely limited [2,17,20]. In addition, the ethical implications regarding animal welfare when using best practices are highly relevant [21]. Minimizing the negative impacts of different research practices on wildlife also improves the quality of the results obtained by assessing responses that more closely resemble those occurring under natural conditions [22].

Fecal metabolites of glucocorticoids (FGCs) provide information on hormone levels over time, as opposed to the instantaneous information obtained when circulating GCs are quantified in plasma [17,23,24]. In addition, these metabolites allow the study of HPA activity and its reactivity to various environmental perturbations [25]. The analysis of GC metabolites does not require the capture of animals, allowing repeated sampling over different periods [24]. However, there are some important factors to consider, starting with the fact that the target hormone is not measured directly. Indeed, its metabolites are measured after processing them in the liver and gut (where a changing microbiota plays an important role), with a delay in appearance in feces depending on gut transit time [12,20,26,27]. Validation of hormone assays must then ensure that metabolite levels reflect adrenal activity under a variety of environmental and social stressors, and provide information on potential sex differences and reproductive status in hormone metabolism [19]. To improve the accuracy of hormone dosages obtained from fecal samples, it is important not only to avoid contamination by urine, which significantly increases GC metabolites [19], but also to consider how metabolites change over time since defecation and after exposure to various environmental factors such as temperature and humidity [28,29]. Therefore, it is important to take proper care when collecting fecal samples.

The increasing number of studies using fecal hormone metabolite assays in natural populations highlights the importance of this technique. However, since GC metabolites are excreted in a species-specific manner [24,26], it is critical to know the time delay between activation of the HPA axis and subsequent reflection of GCs in feces for wild animals [17]. Hence, careful validation is required to ensure that the immunoassay used allows their adequate quantification [26]. As recommended, it is essential to carry out physiological and biological validation (including different conditions and both natural and artificially induced GC levels) to assess the sensitivity of the assay in reflecting relevant stress responses of individuals, both in the field and under controlled laboratory conditions. This approach requires non-invasive sampling and/or extended monitoring periods [20]. In contrast, plasma doses of GCs may prove superior for assessing acute stress and short-term responses. Therefore, it is crucial to assess the impact of this invasive method due to its influence on the subsequent state of individuals and data quality [30,31].

The Talas tuco-tuco (*Ctenomys talarum*) is a solitary, herbivorous, subterranean rodent distributed along the coastal grasslands of Buenos Aires Province (Argentina). Adult individuals are sexually dimorphic in body mass, with males being larger than females [32]. Both sexes occupy exclusive closed galleries parallel to the soil surface [33] consisting of a main tunnel and a variable number of lateral branches and feeding tunnels [34]. Most of their daily activities take place inside their burrows, but they venture above ground for short time periods to collect the aerial vegetative portion of grasses for later consumption below ground [35]. This species is a very interesting model to study the ecophysiology of the HPA axis in rodents. Using plasmatic samples of GCs, it has been found that cortisol and corticosterone show different patterns of variation in the wild and in captivity, and are differentially affected by environmental stimuli [36,37]. In the wild, cortisol levels are significantly higher in females, but not corticosterone [38]. In addition, the levels of both GCs decreased significantly once in the laboratory. This condition is more pronounced in females, especially for cortisol, resulting in subtle or no differences between the sexes [38]. Despite this effect of captivity, individuals of both sexes showed responsiveness to different challenges. Cortisol increases during the ACTH (adrenocorticotropic hormone, precursor of GCs in the HPA cascade) stimulation test [38] and decreases during the DEX (dexamethasone) suppression test [39]. In addition, increases were detected after immobilization [36,37,40], fasting and poor diet quality [39], and simulated infection challenge with LPS (lipopolysaccharide; [41]). Furthermore, when the relationship between personality traits and the physiological response to immobilization stress was examined, it was found that more active and bolder individuals had lower baseline plasma cortisol levels [42].

*C. talarum* has been studied in a wide range of physiological, ecological, and behavioral aspects related to the particular selective pressures associated with the subterranean habitat [43]. The availability of a new tool, such as the quantification of FGCs, will allow these studies to be deepened, both in the field and under controlled conditions in the laboratory. In addition, these organisms live in coastal grasslands, which are subject to increasing anthropogenic impacts as a result of tourism and agronomic activities. Therefore, evaluating responses to combinations of different stressors is currently an important objective.

The main objectives of the present study were:

(1) To evaluate the effect of blood sampling on plasma cortisol concentrations in *C. talarum*. Glucose and the neutrophil-to-lymphocyte ratio (N/L) were also measured to assess whether these parameters accompanied the cortisol variation. (2) To evaluate the suitability of an enzyme immunoassay (EIA), previously validated for cortisol in plasma in *C. talarum,* for measuring FGCs. While assessing the usefulness of fecal metabolites for evaluating adrenal activity, we measured the physiological responses of this rodent under various biologically relevant conditions: (a) Field capture, transportation and captivity, (b) Adrenocorticotropic hormone (ACTH) stimulation test, and (c) Immobilization. Furthermore, (d) differences in FGCs were quantified by sex and reproductive status in wild individuals, and (e) we assessed whether behavior and physiological response correlated by determining whether there were differences in FGC levels between bold and shy individuals.

We predicted that (1) plasma cortisol would increase as a result of blood sampling and that glucose and N/L would accompany such changes. We also expected (2) FGC concentrations in feces obtained from animals in the field to increase due to (a) the stress of capture, transportation, and novel captivity conditions. (b) Higher levels of FGCs are expected in individuals injected with ACTH or (c) subjected to immobilization, but no changes are expected in those injected with saline. (d) Reproduction is expected to affect FGC levels in individuals in the wild, with higher levels in those individuals subjected to higher energy demands as occurs during the breeding season. Finally, (e) lower adrenal activity is expected to be positively associated with boldness and motor activity.

## 2. Materials and Methods

### 2.1. Subjects and Housing

Adult individuals of *C. talarum* (*n* = 41 males, *n* = 47 females) were captured in August–October 2021 (during the species’ reproductive season, *n* = 31 males, *n* = 32 females) and March–April 2022 (non-reproductive season, *n* = 10 males, *n* = 15 females) in Mar Azul, Buenos Aires Province, Argentina (37°34′ S, 57°03′ W) using tubular wire mesh traps (10 cm diameter, 30 cm length) placed at burrow entrances. Females showing signs of lactation (elongated nipples and hairless around the base of them) were immediately returned to their burrows to prevent them from abandoning their dependent young. The remaining females had open or resealed vaginal membrane and were probably in the early stages of pregnancy. Individuals were captured between morning and early afternoon and transported over 100 km in opaque PVC tubes conditioned with paper towels and green grass cut near burrows, to provide shelter, food, and hydration. Upon arrival at our laboratory, tuco-tucos were individually housed in plastic cages (30 cm × 40 cm × 25 cm) with wood shavings, and half a flower pot for shelter. Fresh food—sweet potatoes and mixed grasses—was provided daily to ensure water supply. The animal room was provided with an automatically controlled temperature (23 ± 1 °C) and natural light. At the end of the experiments, the animals were returned to the field in good physical condition.

### 2.2. Blood Collection and Stress Markers

We investigated the effect of blood collection on plasma cortisol levels and the time to return to baseline levels. To achieve this objective we used adult individuals of *C. talarum* (*n* = 20 males, *n* = 19 females; mean ± se body weight: 174.7 ± 6.1 g and 135.1 ± 3.1 g, respectively) captured from August to October 2021, during the reproductive season, and acclimated to captive conditions for ~10 days. Blood samples were obtained within 3 min (detailed in [36]), using a brief anesthesia with inhaled isoflurane. For each individual, two blood samples (~100 µL) were obtained from the orbital sinus using a syringe (SensiMedical, Lifelong Meditech Private Limited, Haryana, India) with a flexible plastic tube (MCM, Jiangsu Jichun Medical Devices Co, Ltd., Jiangsu, China) connected to a heparinized microcapillary tube; group (1) T0 (time of first blood sample) and T30 min (time of the second blood sample) (6 males, 7 females), group (2) T0 and T60 min (7 males, 7 females), group (3) T0 and T120 min (7 males, 5 females). A few days after blood sampling, the animals were released in good condition at the capture site. Plasma cortisol samples were analyzed using a DRG ™ kit (Cortisol ELISA EIA-1887, solid phase enzyme-linked immunosorbent assay). This assay can measure cortisol levels up to 800 ng/mL, with a limit of detection of 2.5 ng/mL and intra- and inter-assay coefficients of variation (CVs) of 7.3% and 12%, respectively (see validation in [39]). In addition to cortisol, blood glucose, and N/L were measured as these parameters usually change in response to stress [1,44] and have been used in previous studies in *C. talarum* (e.g., [36,39,41,45,46]). Blood glucose concentration was measured immediately after each blood collection using a glucometer (Accu-Chek Performa, system range: 20–600 mg/dL, Accu-Chek Performa, Roche, Mannheim, Germany), as stress-induced GC secretion is expected to result in the mobilization of energy reserves and thus elevated blood glucose [9]. The number of lymphocytes, neutrophils, eosinophils, basophils and monocytes was determined under a microscope(Olympus CX31RBSFA, Olympus Corporation, Tokyo Japan) at 450× on May–Grunwald–Giemsa-stained blood smears. All cell types were counted in a total of 200 leukocytes and then the N/L ratio was calculated. The N/L ratio is a stress parameter that regularly increases with GC secretion, especially in the context of chronic stress [47,48,49]. In *C. talarum*, increases in the N/L ratio were associated with stressful situations such as short-term immobilization [36] and chronic stress (food restriction, [46]).

### 2.3. Feces Collection

Feces collected immediately after capturing individuals in the field were used to compare the levels of metabolites of GC for both sexes during the reproductive season (from August to October 2021; *n* = 11 males, *n* = 13 females; mean ± se body weight: 178.5 ± 2.9 g and 124.8 ± 2.9 g, respectively) and the non-reproductive season (March to April 2022; *n* = 10 males, *n* = 15 females; 164.7 ± 5.5 g and 119.2 ± 2.8 g). Most of these individuals were released at the site of capture after feces collection, except for 11 animals of each sex captured during the reproductive season that were transported to the laboratory for validation experiments (see below). As handling individuals usually induces defecation, fresh feces without urine contamination were collected, and then stored in a freezer (−20 °C) until FGC extraction and quantification.

Captive tuco-tucos were individually housed in cages as described above, but with a wire mesh floor. Fecal samples (range 2–10 fecal pellets) were collected at each sampling time (see below) from the cage floor, which was covered with paper towels. Only feces not contaminated with urine were collected directly using tweezers, placed in 5 mL vials and immediately frozen at −20 °C until further analysis. After each sample collection, the tweezers were carefully cleaned with ethanol, and the paper towels were completely replaced. In addition, the grass was removed, and a new bunch was provided in order to avoid the possibility that some feces would remain attached to the plant material and be collected in the following period. Fecal production was not quantified in this study because *C. talarum* is coprophagous, eating feces collected from the ground but also directly from the anus [50]. In addition, feces production and reingestion vary depending on the quality of the available food [50,51].

### 2.4. Enzyme Immunoassay Validation for FGCs

One group of *C. talarum* individuals (N = 6 males and N = 6 females; 171.1 ± 6.4 g and 120.8 ± 3.4 g) remained in captivity for 4 weeks and all animals were used to evaluate: (a) the effect of capture, transportation and captive conditions (first week), (b) ACTH challenge and saline exposure, and (c) behavior (see below). During the second and third weeks, half of the animals were initially assigned to the saline or treatment group with ACTH and then reversed the following week. Feces collection began at 16:00 h (local time) when the animals arrived at the laboratory and continued until 22:00 h (every two hours) on the first day. On the second day, samples were collected at 8:00, 12:00, 16:00 and 20:00 h, on the third and fourth days twice (8:00 and 20:00 h), and on the fifth day at 8:00 h. On the following two days, the animals were given food only and the cages were cleaned (paper towels and grasses were changed at night). For the second and third weeks, feces were collected according to the following schedule: from 8:00 to 20:00 h (every 4 h) on the first day (D-1; pre-treatment day) to know the baseline levels of FGCs, from 8:00 to 22:00 h (every 2 h) on the second day (D1; ACTH or saline injected at 8:00), from 8:00 to 20:00 h (every 4 h) on the third day, and twice on the fourth day (8:00 to 20:00 h). The following three days, the cages were cleaned, and food was provided. The experimental design allows the comparison of the same individuals under different conditions, which serve as their own control, as recommended by [20]. The temporal sampling scheme responds to the fact that daily activity in *C. talarum* is mostly concentrated in the diurnal period, as shown in field and captivity studies [52,53]. Three individuals (one male and two females) were not included in the data analysis for ACTH challenge and saline exposure because feces could not be obtained for all sampling periods, possibly due to low fecal production or increased reingestion.

For the ACTH challenge, synthetic ACTH (Acthel©, GP Pharm, Buenos Aires, Argentina) was injected intramuscularly on day 2 (for the second or third week, see above) at a dose of 4 UI/kg, according to a previous study in *C. talarum* [38]. The injection volume was the same for all animals (100 µL) because isotonic saline was used to compensate for differences in body mass. Saline was used for control individuals (100 µL), as an injection alone can also affect GCs levels [54].

Another group of individuals (N = 5 males and N = 5 females; 187.4 ± 8.4 g and 131.7 ± 5.2 g) was used to assess changes in FGCs due to immobilization stress. These animals remained in captivity for one week to acclimatize to the laboratory conditions. Then, on the first day of the second week, individuals were immobilized with a grid for 1 min at 8:00 h and feces were collected every three hours (pre-test sample: 8:00 h, post-immobilization samples: 11:00, 14:00, 17:00 and 20:00 h). The following day, feces were collected only at 8:00 h.

Since feces handling before analysis can affect the measurements obtained, we evaluated whether the interval between fecal sample collection and freezing affects FGC concentrations. Large fecal samples, each comprising at least 15 units, were collected immediately after defecation from five individuals (two males and three females) and divided into five subsamples. For each individual, the first subsample was frozen at −20 °C immediately after collection. The remaining subsamples were maintained at 23 °C and subsequently frozen after 2, 4, 12, or 24 h. This approach controlled for the potential impact of time exposure to room temperature on fecal integrity, consistent with the collection periods used in the experimental treatments.

### 2.5. Extraction of FGC Metabolites and Enzyme Immunoassay

Glucocorticoid metabolites were extracted according to the protocol of [55]. Frozen fecal samples (−20 °C) were dried at 70 °C to constant weight (24 h) and then ground in a mortar to a fine powder. A sample of 50 mg was placed in a test tube containing 1 mL of 80% methanol, gently vortexed for 10 s to homogenize, shaken at 1400 rpm for 30 min and then centrifuged at 2500× *g* for 20 min. The supernatant was transferred to Eppendorf tubes and stored at −20 °C until analysis.

The quantification of GCs by EIA is an effective alternative to radioimmunoassay (RIA), primarily due to its high performance and non-radioactive nature (e.g., [56]). While EIAs were originally developed to detect the parent hormone (e.g., cortisol or corticosterone) in plasma samples, it has been recognized that the antibody may have sufficient cross-reactivity to measure metabolized forms (e.g., [54,56,57]). EIAs using specific antibodies against GC metabolites (e.g., 5α-pregnane-3β,11β,21-triol-20β-one) were subsequently developed to increase reactivity with metabolized forms [55,58]. However, it has been reported that both types of EIA perform similarly in the quantification of GC metabolites [59]. Furthermore, EIAs for cortisol have been shown to be more sensitive than those designed for cortisol metabolites in the quantification of metabolites; even HPLC analyses have demonstrated that cortisol is almost completely metabolized in feces [60].

The cortisol DRG^®^ EIA kit (#1887, DRG Instruments GmbH, Marburg, Germany) has previously been validated for plasma cortisol in *C. talarum* [39], but this is the first time we have used it for fecal cortisol metabolites. While cross-reactivity has been analyzed for corticosterone in plasma samples, metabolite characterization is not available for fecal samples. Therefore, for the sake of conservatism, we refer to products quantified in feces in this study as FGCs. As reported for other steroid hormone assays in *C. talarum* [36,37,39,42], appropriate validation was performed, including assessment of parallelism, accuracy, and precision [61]. Similarly to plasma samples, parallelism was assessed for FGC extracts by analyzing a series of dilutions using a 0 calibrator (1:1, 1:5, 1:10, 1:15, 1:20, 1:25) and obtaining a slope parallel to that of the standard cortisol curve provided by the manufacturer (*t*-test for equal slopes, *tobs* = 0.667, *df* = 10, *p* = 0.52). Accuracy was not assessed for FGCs because it is incorrect to determine the recovery of already known amounts of the target hormone, i.e., cortisol, which was added to aliquots of metabolites of cortisol samples before assays [62]. Precision was assessed by calculating intra- and inter-assay CVs. Intra-assay CVs were 8.72% (40% binding) and 6.91% (17% binding) calculated from 8 replicate samples assayed in the same plate for each case. In comparison, inter-assay CVs were 10.09% and 15.7%, obtained by running the same two samples in 5 different plates. Throughout the study, FGC levels are expressed as ng/g dry feces.

### 2.6. Behavior

During the fourth week in captivity and after completion of the ACTH and saline experiments, the behavior of individuals (4 females, 5 males) was assessed using three test apparatuses: Open Field, and Open Field with Predator Odor and Escape Tube. For the present study, only those behaviors most indicative of personality traits previously observed in *C. talarum* were selected [42,63]. Animals rested for 1 day between tests. Subjects were allowed to habituate to a dark Plexiglas cage (45 cm × 30 cm × 30 cm) for 1 h before opening the door at the beginning of each Open Field test. Subjects entered the test apparatus at will, with a maximum waiting time of 30 min. Activity was assessed by allowing animals to freely explore an Open Field [64]. Each individual was placed in the experimental home cage, which was connected to the 1 m^2^ Open Field by a 10 cm tunnel. The test was recorded with a zenithal camera from the first entry of the subject and for 10 min. As a measure of activity, the total distance traveled by the subject in the Open Field (quantified as the number of lines crossed by the subject on the 10 × 10 cm grid) was recorded. Boldness was assessed using an Open Field test with a predatory stimulus. The odor of a male domestic cat was provided as the odor source (following previous physiological and behavioral studies of antipredatory responses in *C. talarum*; [40,63]. A 6 × 6 cm piece of cloth impregnated with fur odor (obtained by allowing a cat to lie on it for a week) was placed in a Petri dish and covered with wire mesh. Boldness was measured as the total time individuals spent in the Open Field quadrant containing the predator odor sample. Each trial lasted 10 min. The Open Field activity data of one male could not be recorded because it did not leave its home cage. Finally, we tested escape speed by encouraging individuals to flee from predation risk (adapted from [65,66]). Given the subterranean habits of *C. talarum*, we used a 1.5 m PVC tube (10 cm diameter) with a transparent end to allow viewing of the subject, and sandpaper flooring to ensure adequate grip. Subjects were removed from the home cage and released facing the open end by the experimenter, which acted as a risk stimulus. We recorded the time it took for the subjects to reach the transparent closed end of the tube using a stopwatch. The shortest time taken by a subject in three trials was measured as the escape speed or “sprint”.

### 2.7. Statistical Procedures

Plasmatic cortisol, blood glucose, and N/L ratios were analyzed with SigmaPlot (Version 11.0) software using general linear models (GLM) with sex and time as factors (the last one as a repeated, within-subject factor). Normality and homoscedasticity were previously tested with Shapiro-Wilks and Levene tests, respectively. Only for N/L, a log10 transformation was required to meet the normality assumption. The FGCs data were analyzed in R [67] using RStudio (Version 4.5.0 (R), 4.1.2). The FGC levels detected in feces collected from individuals in the field and during the first 5 days in captivity were analyzed using general linear mixed models (GLMM) with de “nlme” package [68]. The FGC level was considered as the response variable, while time and sex were treated as fixed factors, and individuals as repeated measures. In addition, we used GLMM for the analysis of ACTH challenge and saline exposure. Variance modeling using ‘varident’ was required to achieve normality and homoscedasticity in the case of ACTH data. For the pre-test period, baseline levels of FGCs (response variable) were analyzed to determine if they varied throughout the day (samples D-1: 8:00 h, 12:00 h, 16:00 h, 20:00 h, and D1 8:00 h). The effect of ACTH or saline treatment on FGC levels in comparison to the sample immediately before injection were independently analyzed as a way to simplify the analysis [57]. Time and sex were considered as fixed factors and individuals as repeated measures. Animal ID was included in the analyses as a random effect to account for inter-individual variability, and its significance was assessed by comparing models with and without tuco-tuco identity using likelihood ratio tests (LRT; [69]). The baseline level of FGCs in feces obtained during the ACTH stimulation test (time of ACTH injection) and the peaks detected (maximum increase after ACTH injection), regardless of the time at which they occurred, were analyzed by two-way RM ANOVA. The same general analysis scheme (pre-treatment sample versus post-treatment samples) used for ACTH and saline was applied to evaluate the physiological effect of immobilization. One-way RM ANOVA was used to test whether the time between fecal collection and freezing affected FGC levels. To test for differences in FGC concentrations during the reproductive and non-reproductive seasons we used a GLM with the interaction between sex and season, using an −0.5 exponential transformation to achieve homoscedasticity. Pearson correlations were performed among each behavioral variable recorded: sprint (sec), total distance traveled in the Open Field (number of crossed lines in the grid), and time spent (sec) in the predator-odor quadrant of the Open Field, and the levels of FGCs in feces obtained in the field and during the ACTH stimulation test: baseline (time of ACTH injection), peak (maximum increase after ACTH injection), percentage increase (peak value relative to baseline), and time (h) at which the peak of FGCs was detected. To control for type I error, the standard Bonferroni correction was used [70]; the alpha level was divided by the number of correlations calculated (0.05/15) and those with probabilities of 0.003 or less were considered statistically significant.

Results are shown as mean ± standard error (SE) and were considered significant at *p* < 0.05.

## 3. Results

### 3.1. Stress Response to Blood Collection

**Figure 1:**
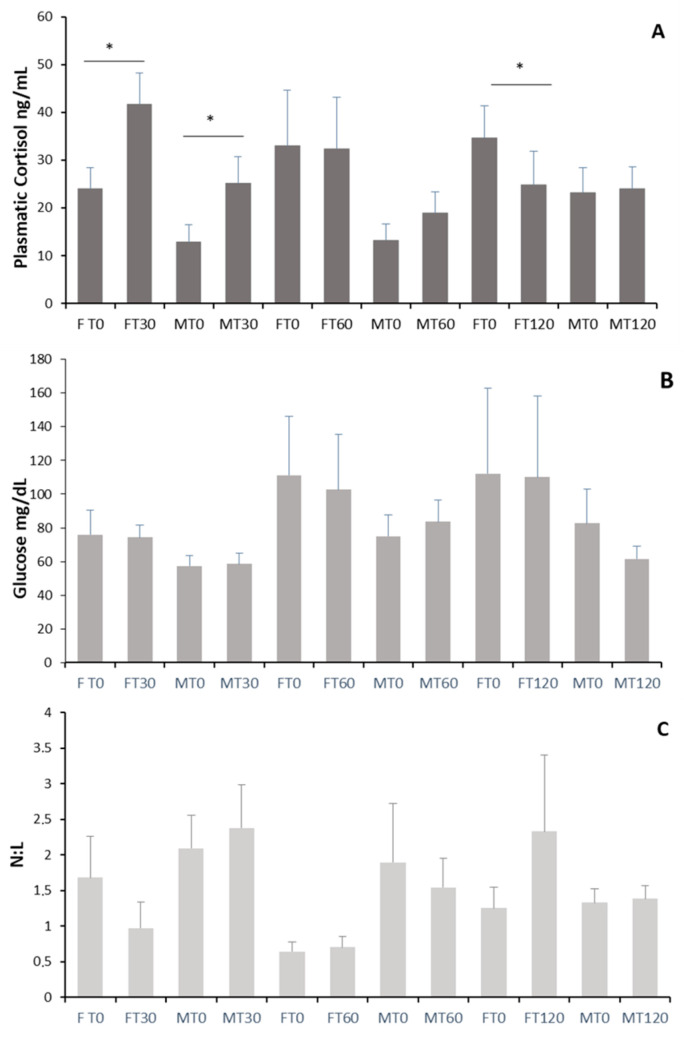
Effect of blood sampling measured in three groups of *Ctenomys talarum* individuals. Blood was collected twice, at T0 and T30 min (7 females, 6 males), at T0 and T60 min (7 females, 7 males) and at T0 and T120 min (5 females, 7 males), for which mean ± SE (**A**) Cortisol (ng/mL), (**B**) Glucose (mg/dL) and (**C**) N/L was determined. Statistical differences (*p* < 0.05) between groups are indicated (*).

Plasma cortisol at T0 was higher in females (30.113 ± 4.475 ng/mL) than in males (16.592 ± 2.57 ng/mL; *t* = 2.525, *df* = 37, *p* = 0.0016). This GC level (Figure 1A) increased significantly 30 min after the first blood sample (F_1,25_ = 21.36, *p* < 0.001), but no differences were found for sex (F_1,25_ = 4.108, *p* = 0.068) or the interaction between sex and time (F_1,25_ = 0.694, *p* = 0.423). One hour after the first blood sample, no differences were found for time (F_1,27_ = 0.147, *p* = 0.708), sex (F_1,27_ = 0.197, *p* = 0.665), or the interaction between both factors (F_1,27_ = 3.634, *p* = 0.081). Similarly, no differences in cortisol levels (F_1,23_ = 4.114, *p* = 0.07) or in relation to sex (F_1,23_ = 0.616, *p* = 0.451) were found 120 min after blood sampling, but an interaction between both factors was found (F_1,23_ = 5.687, *p* = 0.038), as females had slightly lower cortisol levels at the second sampling (Holm–Sidak, all pairwise multiple comparisons; females T0 vs. T120, *p* < 0.05). Blood glucose levels (Figure 1B) did not accompany variations in plasma cortisol. No changes in blood glucose were observed 30 min after blood sampling (F_1,25_ = 0.0017, *p* = 0.968), nor were differences associated with sex (F_1,25_ = 1.79, *p* = 0.208) or the interaction between the two factors (F_1,25_ = 0.078, *p* = 0.784). At 60 min, no changes in glucose levels were detected (F_1,27_ = 0.000, *p* = 1), neither by sex (F_1,27_ = 0.59, *p* = 0.457), but an interaction between the two factors was detected (F_1,27_ = 5.047, *p* = 0.044), although neither comparison was significant (Holm–Sidak All Pairwise Multiple Comparison Procedures; *p* > 0.05). Again, at 120 min, no effects of time (F_1,23_ = 1.642, *p* = 0.229), sex (F_1,23_ = 0.786, *p* = 0.396), or the interaction between both factors (F_1,23_ = 1.124, *p* = 0.314) were found. The N/L ratio (Figure 1C) did not show significant changes associated with blood sampling at any of the time points analyzed, nor did it depend on sex or the interaction between the two factors (T0–T30, time: F _1,25_ = 0.822, *p* = 0.384, sex: F_1,25_ = 1.751, *p* = 0.213, time × sex: F_1,25_ = 4.472, *p* = 0.058; T0–T60, time: F _1,27_ = 0.0006, *p* = 0.98, sex: F_1,27_ = 3.849, *p* = 0.073, time × sex: F_1,27_ = 0.161, *p* = 0.695; T0–T120, time: F_1,23_ = 2.115, *p* = 0.177, sex: F_1,23_ = 0.062, *p* = 0.808, time × sex: F_1,23_ = 1.111, *p* = 0.317).

### 3.2. Enzyme Immunoassay Validation for FGCs

#### 3.2.1. Effect of Capture, Transportation and Captivity

**Figure 2:**
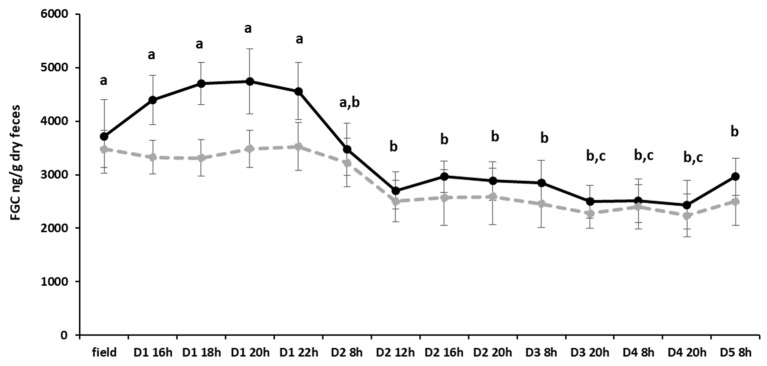
Mean ± SE of fecal glucocorticoid metabolites (FGC, ng/g dry feces) in males and females (*n* = 6 for each sex; males: black line, females: dashed gray line) determined in feces collected in the field and during the first 5 days of captivity in the laboratory. Different letters indicate statistically significant differences (*p* < 0.05) between time periods sampled for both sexes combined.

Levels of FGCs were analyzed for individuals from the field condition and during the first five days in captivity. Differences were found over time (GLMM, F_13,143_ = 13.657, *p* < 0.0001), but not for sex (F_1,10_ = 1.240, *p* = 0.291) and its interaction with time (F_13,130_ = 1.564, *p* = 0.103). As can be seen in Figure 2, FGC levels increased during the first day in the laboratory, with higher levels in males than females. However, the significant individual variation (LRT = 117.33, *df* = 1, *p* < 0.0001) did not allow us to detect statistical differences in the comparison between sexes. However, it is possible to verify that FGC values on the second day of captivity and thereafter were lower than in the wild and on the first day of captivity (Single-step Method for Multiple comparisons *p* < 0.05).

#### 3.2.2. ACTH Challenge Test

**Figure 3:**
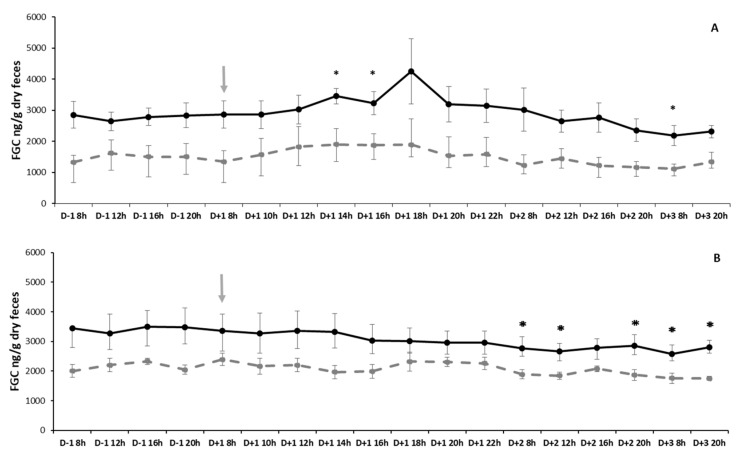
Mean ± SE of fecal glucocorticoid metabolites (FGC, ng/g dry feces) in males (*n* = 5; solid black line) and females (*n* = 4; dashed gray line) determined in feces collected during the ACTH stimulation test (**A**) and control with saline (**B**). The injection time is marked by the arrow. * Indicate statistically significant differences (*p* < 0.05) between time of injection and subsequent time periods sampled for both sexes combined.

The test of adrenal stimulation with ACTH showed that the variation in FGCs was affected by time (GLMM, F_17,136_ = 7.223, *p* < 0.0001) and sex (F_1,7_ = 7.108, *p* = 0.032), but the interaction sex and time was not significant (F_17,119_ = 0.939, *p* = 0.529). Analysis of FGCs for the pre-stimulation period (since D-1 8 h, 12 h, 16 h, 20 h to D + 1 8 h) allowed us to discard a temporal pattern in a daily cycle (Multiple comparisons of Means, *p* > 0.05). Therefore, comparisons between the injection time and each subsequent time were used to evaluate the effect of ACTH on FGC levels. Significant increases in FGC levels were detected six and eight hours after injection (D + 1 14 h, D + 1 16 h; GLM Multiple comparison tests, z-value = −3.713, *p* = 0.003, z-value = −4.710, *p* < 0.001, respectively), whereas a decrease was detected two days later (D + 3 8 h; GLM Multiple comparison tests, z-value = 2.849, *p* = 0.049). Although in Figure 3A it is possible to identify a peak at D + 1 18 h for males, the individual and sex variance precludes reaching statistically significant differences; the random factor had a significant effect on the model (LRT = 203.99, *df* = 1, *p* < 0.0001). However, when baseline FGCs (time of injection) were compared with the peak value (maximum increase after ACTH injection), significant adrenal stimulation was detected (2193.634 ± 378.679 and 3615.346 ± 678.230 ng/g dry feces for baseline and peak levels, respectively; Mean Two-Way RM ANOVA, F_1,17_ = 15.671, *p* = 0.005). Such difference corresponded to variable percentages of increase in FGCs (167.498 ± 9.462%, range: 134.593–218.752%) and time of response (8 ± 0.74 h, range: 4–10 h). No differences were found for sex (F_1,17_ = 0.089, *p* = 0.774) and the interaction of sex × baseline—peak FGC levels (F_1,17_ = 0.304, *p* = 0.599).

The adrenal stimulation test requires a control test with saline because the injection itself could act as a stressor inducing increments in FGCs. The time factor (GLMM, F_17,136_ = 2.811, *p* < 0.001) was found significant, while sex (F_1,7_ = 4.453, *p* = 0.073) and the interaction between sex and time (F_17,119_ = 0.803, *p* = 0.68) were not. As before, the analysis of the pre-stimulation period (since D-1 8 h, 12 h, 16 h, 20 h to D + 1 8 h) allowed to discard a temporal pattern in a daily cycle in FGCs (Multiple comparisons of Means, *p* > 0.05). Comparisons between the time of saline injection and each subsequent time sampled, showed significant decreases in FGC levels (D + 2 8 h, D + 2 12 h, D + 2 20 h, D + 3 8 h, D + 3 20 h; GLM Multiple comparison tests, z-value = 3.006, *p* = 0.026, z-value = 3.435, *p* = 0.006, z-value = 2.813, *p* = 0.046, z-value = 3.895, *p* = 0.001, z-value = 3.218, *p* = 0.013, respectively). Individual variation contributed significantly to the model variation in the analysis of the saline effect (LRT = 152.81, *df* = 1, *p* < 0.0001).

#### 3.2.3. Immobilization

**Figure 4:**
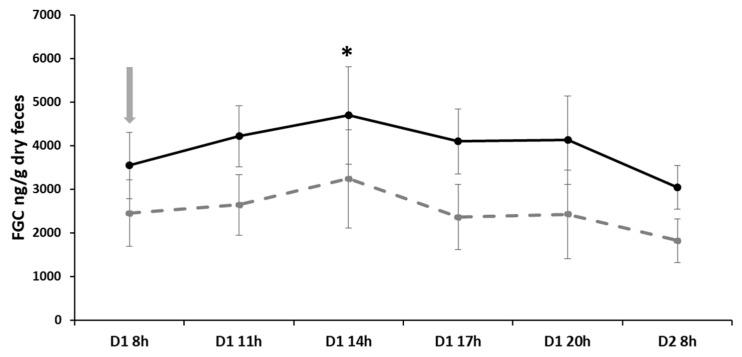
Fecal metabolites of glucocorticoids (FGC, mean ± SE, ng/g dry feces) in *Ctenomys talarum* males (*n* = 5; solid black line) and females (*n* = 5, dashed gray line) following immobilization stress. * Indicate statistically significant differences (*p* < 0.05) between time of immobilization (marked with an arrow) and subsequent periods sampled for both sexes combined.

Immobilization of individuals affected FGCs, with the factors time (F_5,45_ = 5.647, *p* < 0.0004) and sex (F_1,8_ = 5.422, *p* = 0.048) being significant, but not the interaction of sex and time (F_5,40_ = 0.467, *p* = 0.798). Six hours after immobilization, FCG levels increased (Figure 4; GLM Multiple comparison tests, z-value = 3.923, *p* = 0.001). In this statistical model, the random factor had a significant effect (LRT = 36.31, *df* = 1, *p* < 0.0001).

#### 3.2.4. Sample Stability

Sample stability was demonstrated for this study, as FGCs measured in fecal subsamples stored at room temperature for 2, 4, 12, and 24 h were not different from those frozen immediately after animal deposition (T0 = 2904 ± 1941.244, T + 2 = 2894.361 ± 1849.541, T + 4 = 3024.957 ± 2174.104, T + 12 = 2963.324 ± 1913.129, T + 24 = 3120.262 ± 2143.211 ng/g dry feces; One-Way RM ANOVA, F_4,24_ = 1.306, *p* = 0.31).

#### 3.2.5. Sex and Reproductive Seasonality

**Figure 5:**
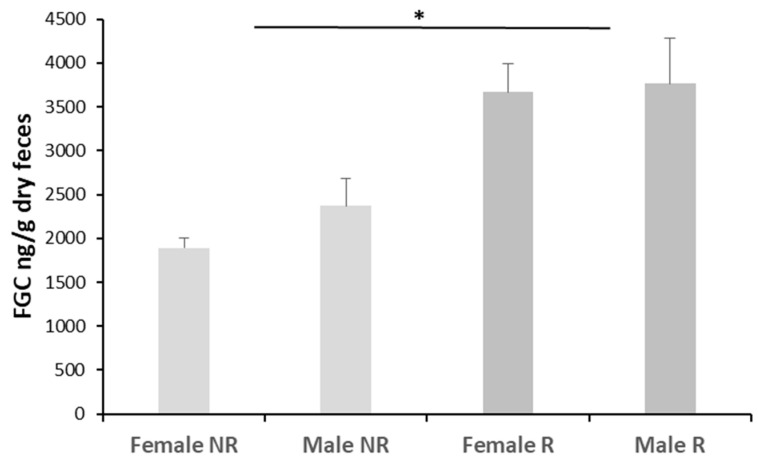
Mean ± SE fecal glucocorticoid metabolite levels (FGC ng/g dry feces) obtained for males and females in the field during the reproductive (R) and non-reproductive (NR) seasons. Sample size in the base of each bar. * Indicate seasonal differences, independent of sex (*p* < 0.001).

The FGCs measured in samples collected from individuals in the field did not differ based on the interaction between sex and season (F_1,45_ = 0.981, *p* = 0.3273) or by sex alone (F_1,45_ = 0.166, *p* = 0.685). In contrast, FGCs differed significantly between reproductive and non-reproductive seasons (Figure 5; F_1,45_ = 26.809, *p* < 0.001; multiple comparisons tests; Tukey contrasts; *t*-value: −4.536, *p* < 0.001).

#### 3.2.6. Behavior

**Figure 6:**
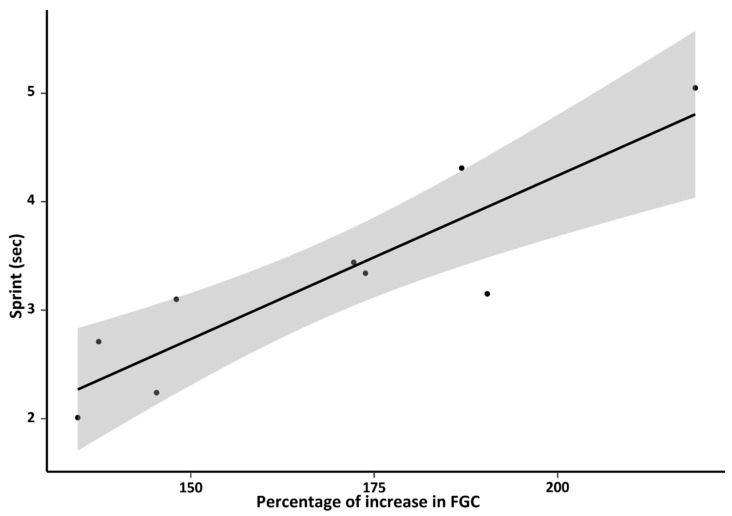
Correlation between the “sprint”, as the shortest time in seconds (out of 3 attempts) required by subjects to run through a 1.5 m tube, and the percentage increase in FGCs after the ACTH challenge test relative to baseline levels. Pearson correlation statistics: *t* = 5.356, *df* = 7, *p* = 0.001, *r*= 0.896.

When assessing the relationship between the recorded behavioral variables-sprint, activity and boldness- and the adrenal activity parameters—FGC levels in feces collected in the field, baseline, peak, percent increase and time of peak detection-, we found that most of the correlations were weak and not significant (Appendix A). However, we found a strong and significant correlation between the sprint and the percent increase in FGCs (Pearson correlation: *t* = 5.356, *df* = 7, *p* = 0.001, *r* = 0.896; Figure 6).

## 4. Discussion

Glucocorticoids serve as effective biomarkers for monitoring stress levels in animals. Obtaining adequate samples requires careful consideration of feasibility and potential impacts on individuals. The present study demonstrated that blood sampling in *C. talarum* increases plasma cortisol concentrations, which return to baseline shortly after sampling, without significant effects on the N/L ratio or glucose levels. To provide a non-invasive alternative for assessing adrenocortical activity, the suitability of a previously validated EIA for measuring plasma cortisol in this species was evaluated for the measurement of fecal glucocorticoid concentrations. The stress response was evidenced during capture and transport, and to a lesser extent during ACTH stimulation and immobilization. A correlation between seasonality and fecal glucocorticoid concentrations was identified, with higher levels observed during the breeding season in both sexes. Lower increases in adrenal activity were also observed in individuals exhibiting faster movement.

### 4.1. Stress Response to Blood Collection

Numerous techniques have been developed to collect blood from small mammals, with varying degrees of discomfort and invasiveness to the individual [30,71]. The procedure used to collect blood samples in *C. talarum* showed effects on plasma cortisol levels at 30 min, which then reversed to be undetectable at 60 min and 120 min, and in the last case, with even lower levels than baseline in females. This suggests that blood sampling acted as a moderate acute stressor, producing a brief increase in GCs, which later returned to baseline by negative feedback. Baseline values are reliable because they were obtained during a time span that does not include the time window of GC release [36]. The observed effects of blood collection are expected because the procedure involves a brief immobilization of the individual, which may be perceived as a predatory event that threatens its survival [40]. Previous studies in *C. talarum* have shown that immobilization induces an increase in GC levels after 30 and 60 min of treatment [36,37]. At the same time, glucose is expected to be released as HPA activation involves energy mobilization associated with the escape response [9,72]. In the present study, the effects on glucose were mild, with a slight increase at 60 min in males. On the other hand, no changes were observed for N/L. Leukocyte redistribution [10] has been proposed as an indicator of stress, especially of a chronic rather than acute type [48]. In agreement with the data obtained in this study, neither changes in glucose levels nor in N/L after a short immobilization have been reported for *C. talarum* [40,45].

Animal welfare implications of research methods are critical. Poor practices are not only ethically questionable but also compromise the quality of the results obtained [21]. Certainly, the use of needles or capillaries (as in this study) for blood collection in vertebrates is rejected a priori. However, the assessment of the impact of this practice is not always well documented. In the case of other vertebrates, such as the wild snake *Natrix tessellata*, it has been shown that the collection of blood does not represent an additional effect—using corticosterone and glucose as stress markers—on the capture of individuals [31]. These results indicate that to make the most appropriate decisions regarding the type of sample to obtain, it is very important to evaluate with adequate experimental designs the different blood sampling procedures and their impact on the endocrine stress response.

### 4.2. Enzyme Immunoassay Validation for FGCs

#### 4.2.1. Capture, Transportation and Captivity

The stress axis is critical for generating physiological responses to a variety of environmental changes [16]. The effects of habitat on the endocrine stress response have been widely studied (e.g., [2,73]), and human-induced habitat alterations elicited particular interest (e.g., [74]). A special case of environmental change is captivity. This is a process in which individuals are exposed to multiple potential stressors, both persistent and sequential acute stressors, including capture, transportation, and placement in a specialized enclosure where they are conditioned to novel environments, i.e., boxes of different sizes, food, shelter, thermal conditions, and light cycles [21]. Understanding the course of the adaptation period to captivity, and the physiological and behavioral adaptations that occur, allows us to expand our knowledge of the adaptability of organisms, the ethical aspects of handling captive animals, the limitations of the data obtained, and how to extrapolate to natural conditions [22,75]. *C. talarum* individuals were affected by capture, transportation, and captivity. Once individuals arrived at the laboratory, GC metabolites showed an increase during the first day of stay, especially in males. However, these results should be treated with caution as the inter-individual variance did not allow us to detect statistical differences. During the following days, FGC levels decreased compared to those in the field and the first day in captivity. Previous studies have shown that the capture technique used does not induce an increase in plasma cortisol for at least a 20 min lapse in the trap [36], but that N/L increases in newly arrived individuals compared to field values [45]. Notably, plasma cortisol decreased in captivity and was more pronounced in females [38]. The available evidence on the effects of captivity does not follow a simple pattern. In some species, FGCs are elevated for the first few days in the novel environment and then return to field levels, which has been interpreted as acclimation to captivity (e.g., Richardson’s ground squirrel, *Urocitellus richardsonii*, [76]). In other cases, corticosterone metabolites were lower and less variable in captivity than in the field (Wood mice, *Apodemus sylvaticus*, [77]). Reduced FGC levels in captivity in *A. sylvaticus* [77] and *Ctenomys sociabilis* [78] have been interpreted as a consequence of favorable conditions, i.e., availability of food and shelter and lack of predation risk. However, it should be noted that these low FGCs may reflect chronic stress. Although chronic stress is often characterized as a state of persistently high GCs, Ref. [79] suggested that the pattern is not clear and that a decrease in their values is also possible. Long-term stressors can cause changes in the regulation of GCs. However, the specific part of the response (baseline levels, stress-induced concentrations, and negative feedback), as well as its magnitude and direction, may vary among species and the challenges they face [79,80]. Therefore, responses to captivity are highly species-specific [81].

#### 4.2.2. ACTH Challenge Test

The ACTH challenge is a pharmacological validation used to measure whether a specific EIA can detect increases in endogenous GCs through their metabolites in feces [26]. The adrenal stimulation test showed that FGCs significantly increased after ACTH injection compared to baseline levels (pre-treatment sample), but no increase was observed after saline injection. Analysis of samples collected the day before the test revealed no diurnal cycle of hormone secretion, which is consistent with the daily activity pattern of *C. talarum*, which alternates periods of movement and rest [52,53]. Significant increases in GC levels were detected 6 and 8 h after ACTH stimulation. A further increase occurs 10 h after injection, particularly in males. However, due to individual variability, statistical significance cannot be achieved. Nonetheless, by analyzing the relationship between the baseline and peak values of each individual, which occurred within the range of 4 to 10 h, adrenal stimulation in both sexes can be confirmed. GCs released after adrenal activation are mainly metabolized in the liver [19]. These metabolites are transported through the gastrointestinal tract for excretion in feces, requiring time depending on the species and type of food [82,83]. The time for GC clearance is not known for *C. talarum*, but gut transit time was calculated for two of the main plant species in their diet, *Panicum racemosum* and *Bromus unioloides*. The results showed that it takes approximately 2–5 h for metabolized products to appear in the feces [50,51]. These estimations are consistent with the time frame of the increase in FGC after ACTH injection. Finally, both the ACTH and saline tests showed a decrease in FGC levels over time, which may explain the effects of chronic stress on the individuals.

The temporal profile of GC metabolites depends on the adrenocortical activity of the individual, but also on steroid metabolism and defecation patterns. As a result, several studies in rodent species have reported highly variable time scales for FGC excretion following stress events or ACTH testing. For example, FGC excretion times were 2 h in deer mice *Peromyscus maniculatus* [59], 4 h in Arctic lemmings *Lemmus trimucronatus* [84], 6 h in *Octodon degus* [85], 8 h in red squirrels *Tammiasciurus hudsonicus* [58], 6–8 h in bank voles *Myodes glareolus* [86], 8 h in eastern chipmunks *Tamias striatus* [60], 6–14 h in rats *Rattus rattus* and *R. norvegicus* [87,88], about 6–10 h in desert gerbils *Gerbillus andersoniallenbyi*, *G. nanus*, and *G. piridium*; [54]), and more than 20 h in Syrian hamsters *Mesocricetus auratus* [89]. For the genus *Ctenomys,* only social species have been studied, and peaks in FGC have been reported at 12 h for *C. opimus* [90] and 16–24 h for *C. sociabilis* [78].

Individual differences play a critical role in studies of HPA axis activity [20]. Model organisms are subject to artificial selection, which can reduce genetic variability and limit observed responses to different challenges. In contrast, in wild organisms, greater variance in stress responses is expected, both from genetic [15] and other prenatal [91] or early developmental [92] origins. In the field, individuals are exposed to a wide range of environmental factors that vary at different scales and can have an impact on HPA axis activity, such as predation events [93]. Although many conditions are standardized under captive conditions, differential responses to confinement may also be important [15]. Therefore, longitudinal studies with FGCs have the advantage that each individual can act as its own control. Other factors such as sex, diet, metabolism and gut microbiota should also be considered as sources of variability in these studies, as they are significant in influencing the composition and concentration of GC metabolites in feces [94].

#### 4.2.3. Immobilization

Immobilization of *C. talarum* individuals resulted in increases in FGC at 6 h post-treatment in both males and females, which is in the temporal range found for ACTH stimulation. It is widely proposed that the stress response is modulated by factors such as the intensity, frequency, and context of perceived challenges, thereby counterbalancing the detrimental effects on fitness that result from excessive or chronic activation of the HPA axis [6,8]. Activation of the stress axis in *C. talarum* occurred following stimulation with cues indicating the presence of a predator, as evidenced by plasma cortisol at 30 min [36,37]. Furthermore, immobilization produced a greater increase in plasma cortisol than exposure to cat odor. This is expected as the effect of a physical stressor is interpreted as a direct risk to survival due to the difficulty of escape [40].

#### 4.2.4. Sample Stability

Assessing the stability of fecal samples is necessary for accurately measuring stress levels using GC metabolites [20]. The basic recommendation to collect and freeze samples immediately after deposition is not always possible, so it is important to know the time periods during which metabolites remain at or near their excretion levels. Although changes in GC metabolites are more likely to occur under field conditions due to the effects of ambient temperature and humidity [28,29], it is also important to consider these changes in studies conducted under controlled laboratory conditions, where samples are collected at different times. In the case of *C. talarum*, feces stored at room temperature for up to 24 h showed no differences in the levels of FGC detected by the EIA kit used. Although it is intuitive to expect that fecal metabolites decay over time, results from different groups of organisms have shown increases, decreases, or no change. For instance, fecal subsamples stored for 5 h at ambient conditions for North American ground squirrels showed no differences from those immediately frozen [58]. Similar results were found for the European Badger (*Meles meles*), a carnivorous mammal, where cortisol metabolite levels remained stable for up to 8 h between feces collection and freezing [95]. In contrast, for the chamois *Rupicapra rupicapra* and red deer *Cervus elaphus*, both ungulates, metabolite levels decreased over time [96]. Additionally, [97] observed an increase in fecal metabolites in alpacas (*Vicugna pacos*) 4 h after defecation. Changes in GC metabolites can occur through the action of microbes that can convert them to other steroids through metabolism or degradation, especially under high humidity conditions [23,98]. However, in addition to the effects of environmental factors, it should be noted that the results of GC dosing depend on the species and the technique used, e.g., [99] reported increases or decreases in FGCs in sheep when using different EIAs.

#### 4.2.5. Sex and Reproduction

Differences in FGC concentrations between the sexes have been widely reported. However, the biological interpretation of this information is not of major relevance. On the one hand, the sexes are known to differ in hormone metabolism, so similar amounts in plasma may result in different levels of metabolites in feces. On the other hand, immunoassays have affinities for specific metabolites, so that dosage results from the combination of both factors [94]. In *C. talarum*, similar amounts of FGCs were detected for both sexes in the field during the reproductive and the non-reproductive season, even though plasma cortisol levels differed between sexes, being higher in females [36,37]. This may be attributed to the fact that the whole metabolite composition does not differ between sexes, or the metabolite composition extracted and reacting with the EIA kit used is the same among them [20]. Moreover, plasma cortisol decreased during captivity, and was more pronounced in females, reducing sex differences [38]. In the case of FGCs, there was a general decrease over time, but this resulted in lower levels in females than in males.

Seasonal changes in reproductive activity are expected to affect GC secretion, as these hormones play an important role in regulating energy balance [100]. Our study found that baseline FGCs varied according to reproductive seasonality in *C. talarum*, with males and females exhibiting higher levels during the reproductive season, which is consistent with the findings reported for plasma cortisol [37]. According to the Energy Mobilization Hypothesis, baseline FGCs are expected to be higher during the reproductive season [100]. In *C. talarum*, males engage in territory and mate defense, while females experience pregnancy, lactation, and maternal care, all of which are energetically demanding processes during reproduction [101,102]. In addition, since GCs have orexigenic effects, higher concentrations may also contribute to coping with the energetic demands of reproduction by stimulating food consumption [103]. It is important to note that the females included in this study were in the early stages of gestation during the reproductive season. Considering that lactation is the most energetically demanding period in *C. talarum* [102], FGCs could be higher for this group. On the other hand, the Preparatory Hypothesis predicts that FGC levels will change based on the probability of encountering stressors [100]. While the subterranean environment provides shelter from predation, surface activity and the associated risk of predation increase during foraging (for both sexes throughout the year) and when males visit female burrows for mating during the reproductive season. Therefore, no identifiable period with a higher incidence of stressors could be proposed. To test this hypothesis, it is necessary to record the changes in the vegetation cover during the seasons, which will serve as a protection against predators. Revealing the effects of reproduction on GCs in free-ranging small mammals is not an easy task. The direction and magnitude of FGC levels do not follow a clear pattern associated with reproductive conditions. For instance, in Eurasian red squirrels, *Sciurus vulgaris*, FGC concentrations were highest in lactating females [104]. Meanwhile, in *T. hudsonicus*, pregnant females exhibited higher FGC levels than lactating and non-breeding individuals, while reproductive conditions in males did not affect FGCs [58]. Additionally, [105] found that FGC decreased from pregnancy to lactation in the small brown myotis (*Myotis lucifugus*).

#### 4.2.6. Behavior and Adrenal Activity

Individuals exhibit diverse coping styles, which are considered different patterns of response to stressors. These styles can be (broadly) categorized as proactive or reactive, arranged along a continuum. According to this perspective, there should be strong correlations between physiological and behavioral responses to stressful conditions. Proactive individuals are expected to be more active, exploratory, and have lower HPA axis activity [15]. However, the evidence in rodents about the link between behavior and HPA reactivity became mixed (see revision [106]), with some studies supporting the initial unidimensional model [15], others showing an opposite-direction correlation, and yet others showing no association [88,107,108,109]. For tuco-tucos we found that motor activity and boldness (both sexes included due to limited sample size) were not associated with parameters of adrenal activity measured by FGCs. Similar results were found for sprint behavior, but interestingly, a positive and strong relationship indicates that individuals moving fastest in a tube have lower increments in adrenal activity. This finding aligns with a previous study in *C. talarum* males subjected to immobilization stress, in which baseline plasma cortisol levels were lower in bolder and more active individuals [42]. Thus, both findings support the idea that animals living in stable environments, such as underground burrows [43], are expected to show a proactive coping style [62]. In its natural environment, an individual’s running performance can affect critical tasks related to survival and reproduction. This includes finding food [110,111], avoiding being eaten by predators [112,113], protecting its territory from coespecificts and acquiring mates [114,115]. Despite underground habitats offer protection and stability, tuco-tucos face predation risk while foraging [35]. Predator exposure leads to increased—but moderated—cortisol and anxiety, suggesting that predation pressure is only partially reduced by their underground lifestyle [40], thus giving the running performance an important role in avoiding and escaping from predators. The results given here, as well as in other studies, suggest that the topic merits further research.

## 5. Conclusions

In this study, we verified that obtaining blood through the procedure used had an effect on the activation of the HPA axis that is reversed in the short term. Therefore, for some research objectives that cannot be resolved using GCs metabolites, it is possible to use this methodology. Furthermore, the EIA used was successfully validated to measure metabolites of GCs in *C. talarum*, detecting increases in FGCs following ACTH stimulation and immobilization, effects of captivity, and reproductive seasonality. Furthermore, we provide evidence of an association between physiological and behavioral responses. Future studies will contribute to elucidate the extent to which this association contributes to individual variability in adrenal response.

Monitoring animals in the wild and those under experimental treatments often involves the application of practices like immobilization, blood sampling, and captivity. Evaluating the physiological and behavioral consequences of these procedures, with particular attention to adrenal axis activation, facilitates evidence-based evaluation of the cost–benefit ratio between data collection and animal welfare. Furthermore, solitary mammals, despite their high conservation concern [116], received less research attention than social species due to observational and logistical challenges. Their lack of group-based ecological buffering may also heighten vulnerability to climate change [117]. *C. talarum* is a wild solitary rodent with hidden habits living in an increasingly anthropized environment. Thus, developing better tools to evaluate the physiological status and responses to different environmental challenges becomes relevant.

## Figures and Tables

**Figure A1:**
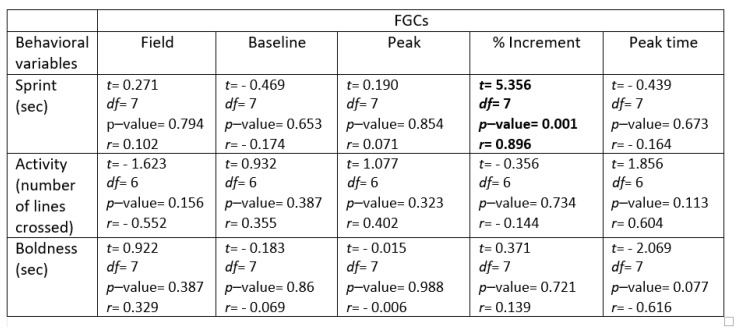
Pearson correlations among behavioral variables and levels of FGCs for *C. talarum* individuals obtained at the time of capture in the field and during the ACTH (adreconocorticotropic hormone) stimulation test: baseline, peak (maximum increase after ACTH injection), percentage of increase (peak value relative to baseline levels) and time (h) at which peak was detected. Statistics for each correlation are shown, with statistically significant values indicated in bold case.

## Data Availability

Request to access the datasets should be directed to the authors.

## Abbreviations

The following abbreviations are used in this manuscript:

HPA	Hypothalamic–pituitary–adrenal axis
SAM	Sympathetic–adrenal–medullary axis
ACTH	Adreconocorticotropic hormone
GCs	Glucocorticoids
FGCs	Fecal metabolite of glucocorticoids
N/L	Neutrophils/ Lymphocytes rate
